# Coronary Artery Disease and Intradialytic Myocardial Ischemia in Hemodialysis: An Exploratory Study Using Intradialytic Imaging

**DOI:** 10.1016/j.xkme.2025.101126

**Published:** 2025-09-24

**Authors:** Lisa Hur, Ali Islam, Jarrin D. Penny, Justin Dorie, Christopher W. McIntyre

**Affiliations:** 1 Department of Medical Biophysics, Western University, London, Ontario, Canada; 2 Department of Diagnostic Radiology, St. Joseph’s Health Care, London, Ontario, Canada; 3 Lilibeth Caberto Kidney Clinical Research Unit, London Health Sciences Centre, London, Ontario, Canada

**Keywords:** Hemodialysis, coronary artery disease, myocardial perfusion, myocardial stunning, intradialytic imaging

## Abstract

**Rationale & Objective:**

Recurrent segmental myocardial ischemia (myocardial stunning) is a well-recognized consequence of hemodialysis leading to fixed contractile deficits and increased morbidity and cardiac mortality. Although epicardial coronary artery disease (CAD) is not a prerequisite, hemodialysis patients commonly have significant arterial plaque burden, and the interaction with established CAD and hemodialysis-induced myocardial ischemic injury is currently unknown.

**Study Design:**

A single-center cross-sectional study.

**Setting & Participants:**

Thirteen patients on maintenance hemodialysis (London, Ontario, and Canada).

**Exposure:**

The presence of significant coronary stenoses as assessed using coronary computed tomography (CT) angiography.

**Outcomes:**

Principal aim was to identify changes in myocardial perfusion and stunning as assessed by CT and echocardiography, respectively.

**Analytical Approach:**

We used CT angiography with intradialytic CT perfusion and echocardiography to evaluate perfusion and ventricular contractile response during dialysis in patients with and without CAD. Coronary artery images were acquired before dialysis (baseline). The CT perfusion scans were conducted at baseline, peak-dialysis stress, and 30 minutes after dialysis to quantify global and segmental perfusion of the left ventricular myocardium. At each timepoint, segmental myocardial stunning was identified as intradialytic development of regional wall motion abnormalities using longitudinal strain analysis from 2D echocardiograms.

**Results:**

Among 13 participants, 3 were identified with asymptomatic CAD. Among the 10 participants with no identifiable CAD, there was a decrease in global myocardial perfusion from baseline to peak dialysis and a recovery to baseline level after dialysis. In asymptomatic CAD participants, the number of myocardial segments experiencing regional wall motion abnormalities was elevated at peak and after dialysis. No significant intradialytic changes in global myocardial perfusion were observed, but the presence of CAD showed additive effects on segmental perfusion and contractile response to HD.

**Limitations:**

This study was an exploratory study with a small sample size.

**Conclusions:**

This study demonstrated hemodialysis-induced circulatory stress, by a reduction in perfusion and development of myocardial stunning in the absence of CAD.

## Plain-Language Summary

Decreased perfusion in the heart muscle is a common consequence of hemodialysis. This often results in reduced contractile function of the heart and is associated with increased morbidity and cardiac-related deaths. Although coronary artery disease is not present in all patients on hemodialysis, many have significant blockage in their arteries, which may worsen the effects of hemodialysis treatment on heart perfusion. We conducted research on 13 hemodialysis participants using multimodal imaging of the heart and confirmed that dialysis-induced reduction in blood flow in the heart muscle that was associated with lower contractility of the heart in patients without coronary artery disease. These effects worsened after the end of the hemodialysis treatment session in patients with coronary artery disease.

Cardiovascular disease is highly prevalent in patients receiving kidney replacement therapy and is a major cause of morbidity and mortality.^1^^,^^2^ Multiple factors prime the myocardial circulation to be at risk of demand ischemia, including uremic cardiomyopathy,^3^ accelerated calcified atherosclerosis,^4^^,^^5^ and arteriosclerosis.^6^ The complex interplay between kidney failure-specific risk factors including chronic volume^7^, ^8^, ^9^ and pressure overload,^10^ neurohormonal activation,^11^ and the accumulation of uremic toxins^12^^,^^13^ are potential pathophysiological mechanisms leading to the development of cardiovascular dysfunction in this population. Uremic toxins accompanying progressive loss of kidney function in chronic kidney disease are protein-bound uremic toxins,^14^^,^^15^ asymmetric and symmetric dimethylarginine,^16^ advanced glycation end products,^17^^,^^18^ phosphate,^19^ klotho,^20^ and fibroblast growth factor-23.^20^ The retention of these toxins triggers a cascade of events that promote and accelerate atherosclerosis, a disorder primarily of the intimal layer of medium-sized arteries and defined by the formation of plaque resulting in the narrowing of the affected vessel.^13^ In conjunction with pronounced calcification because of disturbed calcium-phosphorous homeostasis in patients with kidney failure, the intima-medial layer of the coronary arteries and aorta thickens, resulting in increased arterial stiffness and a loss of vascular wall compliance.^21^^,^^22^ These kidney failure-specific risk factors may increase susceptibility of myocardial ischemia, which can be further exacerbated in hemodialysis (HD) population undergoing repeated hemodynamic stress.^23^

HD treatment has demonstrated efficient removal of toxins and fluid, as well as correcting for electrolyte and acid-base imbalances within a short duration. However, sudden and extreme physiological changes experienced during a single HD treatment are associated with immediate clinical consequences, such as intradialytic hypotension.^24^ As well, patients treated with chronic intermittent HD are subjected to recurrent circulatory stress leading to subclinical organ damage, which can accumulate over time.^25^ This results from multiple vascular beds experiencing ischemia and reduction in oxygen delivery, followed by reperfusion at the end of the HD session, including in the liver,^26^ kidney,^27^ and brain.^28^ HD patients are prone to myocardial ischemia because of the circulatory stress related to HD.^29^, ^30^, ^31^ Endothelial dysfunction and microcirculatory changes can be associated with reduced fractional flow reserve in the coronary circulation, without the presence of significant coronary artery disease (CAD).^30^^,^^32^^,^^33^ Patients with diabetes with kidney failure have been reported to have reduced coronary fractional flow reserve in the absence of angiographically evident CAD.^34^ Patients treated with HD without left anterior descending stenoses had significantly lower coronary flow reserve than than those with normal kidney function.^35^ These findings may be because of structural and functional cardiovascular abnormalities commonly present in this population.^36^ Clinical studies using advanced imaging have demonstrated that CAD is not a prerequisite for HD-related reduction in myocardial perfusion (MP).^37^, ^38^, ^39^, ^40^

Patients treated with HD commonly have widespread and progressive plaque-based epicardial CAD. Other than demonstrating that CAD is not a requirement to develop HD-induced ischemic injury, the interaction between identified coronary plaque and observed HD-induced reduction in myocardial perfusion has not been a subject of direct study. The central question, therefore, is if HD-induced myocardial stunning is common (but not dependent on CAD) and epicardial CAD is widespread is there a potential interaction that may justify investigation into a clinically treatable culprit lesion? The aim of this study was to simultaneously image the coronary circulation, myocardial perfusion, and ventricular segmental contraction during a HD session to directly address this important question for the first time.

## Methods

### Study Design

This study was a single-center cross-sectional study (NCT04036695), conducted on a cohort of patients with kidney failure requiring maintenance HD, approved by the Western University Health Sciences research ethics board and in adherence to the Declaration of Helsinki. Participants who agreed to participate in the study were asked to attend an intradialytic imaging session at St. Joseph Health Centre (London, Canada). During the study, a series of dynamic contrast-enhanced CT images, echocardiography, and blood samples were collected throughout the course of HD: before HD initiation (baseline), at maximal HD-induced circulatory stress (peak HD), and following the recovery phase after the return of blood (post-HD).

### Study Population

Fourteen participants aged 18 years and older were recruited with informed consent from the London Health Science Centre Renal Program (London, Canada). A single participant was removed from analysis because the coronary angiography image could not be acquired at the time of the study visit. In total, 13 participants completed all aspects of the study. Those recruited were required to have received HD for 3 months and more before study enrollment (regardless of known coronary artery status) and undergone HD at least thrice weekly. The exclusion criterion included a history of chronic arrhythmia, being on antiarrhythmic medications, and having implanted cardiac devices such as a pacemaker or cardioverter defibrillator.

### Dialysis Treatment Information

The study visits were conducted midweek, and dialysis treatments were delivered on the Fresenius 5008 dialysis machine using high-flux polysulfone dialyzers by a single operator (JP). HD duration ranged from 3-4 hours. The dialysate composition was prescribed and delivered in accordance with the patient’s individual prescription. Anticoagulation was achieved using low molecular weight heparin, and the dialysate temperature was 36.5 °C for all patients.

### Assessment of Hemodynamic Stability

Blood pressure measurements were acquired on participants’ arrival and periodically throughout the dialysis session until 30 minutes after the end of HD in an upright position.

### Assessment of Coronary Artery Status

Coronary CT angiography (CCTA) was performed at baseline directly before commencing dialysis for noninvasive anatomical assessment of coronary artery stenosis. A smart preparatory feature allowing real-time monitoring of the contrast agent because it enhances the descending aorta at low x-ray dose was used to determine patient-specific parameters. Next, a prospectively electrocardiogram-gated scan was acquired at peak aortic enhancement of contrast during breath hold in the absence of nitroglycerin and β-blockers. The scanning parameters are as follows: DFOV = 25.0 cm; 75%-75% R-R interval; tube voltage = 100 kV; tube current = smart mA 600-700; detector coverage = 160 mm; slice thickness = 0.625 mm; gantry period = 0.28 seconds. Iodine contrast (Isovue 370, 0.7 mL per kilogram of body weight) was administered intravenously at 3-4 mL per second, followed by a set 30 mL saline flush bolus.

An experienced radiologist (AI) reviewed the coronary anatomy for clinically significant stenosis in the following 3 major arteries: (1) right coronary artery, (2) left anterior descending artery, (3) left circumflex (LCx) artery. As per literature, coronary arteries with more than 50% narrowing of the vessel’s diameter were identified to be stenosed lesions with potential clinical hemodynamic significance.^41^, ^42^, ^43^ For each participant, the number of coronary arteries and the specific arteries with the stenosis were recorded. Based on the reported coronary artery status, the participants were divided into 2 arms. Individuals with one or more coronary artery stenoses were distinguished into the CAD+ group, while the remaining participants with no reports of stenosed lesions were distinguished into the CAD- group.

### Dynamic Contrast-Enhanced CT

Prospectively electrocardiogram-gated, dynamic CT images of the heart were acquired at baseline, peak HD stress, and post-HD. To quantify perfusion, iodinated contrast agent (Isovue 370) was delivered during a series of axial scans that repeated every 1-2 heartbeats. The scanner settings for all dynamic CT images acquired for this study were as follows: DFOV = 45.0 cm; 75%-75% R-R interval; tube voltage = 100-120 kV; tube current = 100 mA; detector coverage = 160 mm; slice thickness = 2.5 mm; gantry period = 0.28 seconds. The Johnson-Wilson-Lee model of tracer kinetics was applied following nonrigid image registration (GE proprietary software).

### Quantification of Global MP

For each image set, 7 slices of the left ventricular myocardium taken parallel to the horizontal long axis view were selected (the mid slice, 3 slices posterior, and 3 slices anterior to the selected mid slice) to measure global MP.

### Quantification of Segmental MP

MP was quantified in 16 myocardial segments following reformatting to the standard short axis view. Because the segments are anatomically defined, the amount of MP corresponding to a particular coronary artery responsible for the supply of blood to the particular segments could be measured.

### Assessment of Regional Wall Motion Abnormality

Apical 4-chamber and 2-chamber views of the heart were imaged with echocardiography at baseline, peak HD stress, and post-HD to study the effect of HD on myocardial stunning. Using an offline 2D speckle tracking proprietary software (EchoPac), segmental longitudinal strain (LS) values were calculated for 12 myocardial segments visualized in the 4-chamber and 2-chamber views for all intradialytic timepoints.

Regional wall motion abnormalities (RWMAs) were defined at peak and post-HD by a decrease in LS relative to the baseline strain values, indicating myocardial stunning. For each of the 12 myocardial segments, percent change in LS relative to baseline was calculated at peak HD and post-HD. Of the 12 myocardial segments, those with more than 20% reduction in LS change from baseline were considered to have experienced RWMAs.

### Statistical Analysis

All statistical analyses presented in this paper were performed in GraphPad Prism 9 software (GraphPad Software). Generally, a repeated measure ANOVA was performed for group effect with intradialytic timepoint as a fixed effect. In the assessment of measures with missing data, a linear mixed model was used. The Geisser-Greenhouse correction was applied for sphericity, and either the Sidak or Tukey correction was used for multiple comparisons. A detailed outline of the statistical test performed for each measurement is presented in the supplementary file.

## Results

### Coronary Artery Status

The CCTA findings are summarized in Table 1. Three participants were identified to have multivessel CAD with stenoses more than 50%; the LCx was identified to be the most commonly stenosed in this participant group. One CAD+ participant had stenosed lesions in all 3 of the coronary arteries and was also evaluated with a high calcification score. Ten participants showed no hemodynamically significant lesions that were more than 50% stenosed and one of the participants in this group had high calcification scoring. In total, 39 coronary arteries were imaged across 13 participants; 3 were not evaluable because of poor image quality and were removed from further analysis. Among the 3 participants with CAD, 7 coronary arteries had stenosed lesions with potential for hemodynamic significance (2 right coronary arteries, 2 left anterior descending arteries, and 3 LCx).

**Table 1:** Descriptive Analysis of Coronary Artery Status of Individual Patients

Participant #	RCA	LAD	LCx	Highly Calcified
1	-	-	-	-
2	-	+	+	-
3	-	-	-	-
4	-	-	-	-
5	NE	-	-	-
6	-	-	-	-
7	+	+	+	+
8	-	-	-	+
9	-	-	-	-
10	+	NE	+	-
11	-	-	-	-
12	NE	-	-	-
13	-	-	-	-
Total lesions (%)	15	15	23	15

*Note:* (+) denote lesions with > 50 % stenoses in the respective coronary artery and (-) indicate no stenosed lesions or stenosis ≤ 50 %.

Abbreviations: LAD, left anterior descending artery; LCx, left circumflex artery; NE, not evaluable; RCA, right coronary artery.

### Participants and Dialysis Treatment

Thirteen participants had a complete set of laboratory testing, CCTA, dynamic CT perfusion, and echocardiography image data. The remaining participant was excluded from further analysis because of unsuccessful CCTA. A participant from the CAD− arm was excluded from the RWMA portion of the analysis because of the poor image quality of the echocardiograph. The participants’ demographic information reflected the general HD population (Table 2), and the dialysis prescription and treatment information are detailed in Table 3. None of the patients had previously identified symptomatic CAD.

**Table 2:** Participant Information and Demographics

Characteristics	Absence of CAD (n = 10)	Presence of CAD (n = 3)
Ethnicity: White, n	7 (70%)	3 (100%)
Men, n	6 (60%)	2 (67%)
Age, y	67 ± 12	62 ± 20
BMI, kg/m^2^	34 (27-41)	30 (26-32)
Dialysis vintage (mo)	63 (27-141)	41 (20-73)
Hemodialysis vintage (mo)	53 (16-141)	40 (16-73)
Charlson comorbidity index^a^	7 (4-12)	8 (4-11)
Congestive heart failure	3 (30%)	1 (33%)
**Primary renal diagnosis**		
Hypertension	4 (40%)	0 (0%)
Hypertensive nephrosclerosis	2 (20%)	0 (0%)
Diabetic nephropathy	3 (30%)	3 (100%)
Acute interstitial nephritis	1 (10%)	0 (0%)
IgA nephropathy	2 (20%)	0 (0%)
Current smoker	1 (10%)	0 (0%)
**Medications**		
ACEi/ARB	5 (50%)	0 (0%)
β-Blocker	6 (60%)	1 (33%)
2+ antihypertensive agent	4 (40%)	0 (0%)

Abbreviations: ACEi, angiotensin-converting enzyme inhibitor; ARB, angiotensin receptor blocker; BMI, body mass index; CAD, coronary artery disease.

**Table 3:** Dialysis Prescription and Treatment Information (n = 13)

Dialysis Prescription	Mean ± SD
Treatment time (h)	3.5 ± 0.7
Sodium (mmol/L)	139.0 ± 1.8
Calcium (mmol/L)	1.3 ± 0.1
Bicarbonate (mmol/L)	37.6 ± 2.3
Dialysis flow, Qd (mL/min)	546.0 ± 113.0
**Vascular Access n (%)**	
AVF	6 (46%)
AVG	1 (8%)
CVC	6 (46%)
**Intradialytic Clinical Information**	**Mean** ± **SD**
Weight +gain/−loss (kg ± SD)	1.4 ± 0.7
Pre HD SBP (mm Hg ± SD)	147.0 ± 20.0
Pre HD DBP (mm Hg ± SD)	62.0 ± 18.0
SBP nadir (mm Hg ± SD)	103.0 ± 19.0
DBP nadir (mm Hg ± SD)	52.0 ± 14.0
Kt/V ± SD	1.5 ± 0.3
Min RBV (% ± SD)	85.0 ± 3.5
Mean UFR (mL/h ± SD)	666.0 ± 203.0
Mean UFR/pre weight (mL/kg/h ± SD)	7.6 ± 2.7
Total fluid removed (mL ± SD)	2,548.0 ± 816.0

Abbreviations: AVF, arteriovenous fistula; AVG, arteriovenous graft; CVC, central venous catheter; DBP, diastolic blood pressure; HD, hemodialysis; RBV, relative blood volume; SBP, systolic blood pressure; UFR, ultrafiltration rate.

### Laboratory Testing

**Figure 1:**
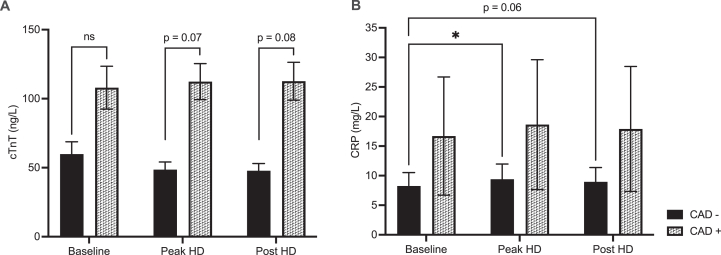
Mean cardiac troponin T (panel A) and C-reactive protein (panel B) in *CAD−* (n = 10) and *CAD+* arm (n = 3) at baseline, peak HD stress, and post-HD. Error bars represent the standard error of the mean. CAD, coronary artery disease; HD, hemodialysis. ^a^∗Denote *P* < 0.03.

Electrolytes demonstrated expected changes resulting from HD (Table 4, Fig S1). Biomarkers of cardiac injury and inflammation analyzed were cardiac troponin T (cTnT) and C-reactive protein (CRP). A mean difference of −48.2 ng/L of cTnT with 95% CI (−125.3 to 28.9) was seen at baseline between CAD− and CAD+ groups (Fig 1A). At peak HD and post-HD, a pattern of increase was demonstrated in cTnT that was relatively higher in the CAD+ group. The mean difference in cTnT at peak HD was −63.7 ng/L with 95% CI (−137.7 to 10.3) and −64.9 ng/L with 95% CI (−145.47 to 15.6) at post-HD timepoint (Fig 1A).

**Table 4:** Mean Plasma Electrolyte Concentration of Those With Significant Changes as a Response to Hemodialysis Treatment

Electrolyte (Conc. ± SD)	Control (n = 13)		
	Baseline	Peak HD	Post-HD
Ionized calcium (mg/dL)	4.37 ± 0.40	4.21 ± 0.40	4.17 ± 0.40
Potassium (mEq/L)	4.62 ± 0.80	3.33 ± 0.40	3.35 ± 0.40
Bicarbonate (mEq/L)	24.50 ± 2.50	30.10 ± 2.20	30.10 ± 2.10
Anion Gap (mEq/L)	16.00 ± 3.70	12.60 ± 2.50	12.10 ± 2.50
Creatinine (mg/dL)	8.28 ± 2.15	2.94 ± 0.97	2.87 ± 1.11
Urea (mg/dL)	108.11 ± 28.23	30.39 ± 9.61	29.13 ± 10.21
Albumin (g/dL)	3.63 ± 0.38	4.15 ± 0.46	4.01 ± 0.46
Calcium (mg/dL)	9.02 ± 1.20	9.14 ± 0.80	8.86 ± 0.80
Magnesium (mg/dL)	2.60 ± 0.49	2.94 ± 0.49	2.24 ± 0.49
Phosphate (mg/dL)	5.48 ± 1.55	2.20 ± 0.31	2.42 ± 0.62
Hemoglobin (g/dL)	10.10 ± 94	11.10 ± 1.10	10.70 ± 1.10
C-reactive protein (mg/L)	7.30 (4.08-16.32)	8.20 (4.74-18.32)	8.10 (4.52-17.51)
Hematocrit (%)	31.00 ± 3.00	34.00 ± 4.00	33.00 ± 3.00

*Note:* C-reactive protein reported as median (lower 95% CL, upper 95% CL).

No significant group (CAD+ vs. CAD−) effect was seen in CRP levels across timepoints. However, within the CAD− group, there was an increase in CRP between baseline and peak HD a median value of 5.4 mg/L (IQR, 2.3-15.0 mg/L) and a median value of 6.2 mg/L (IQR, 2.4-17.4 mg/L), respectively, Fig 1B).

### Systemic Hemodynamic Stability

**Figure 2:**
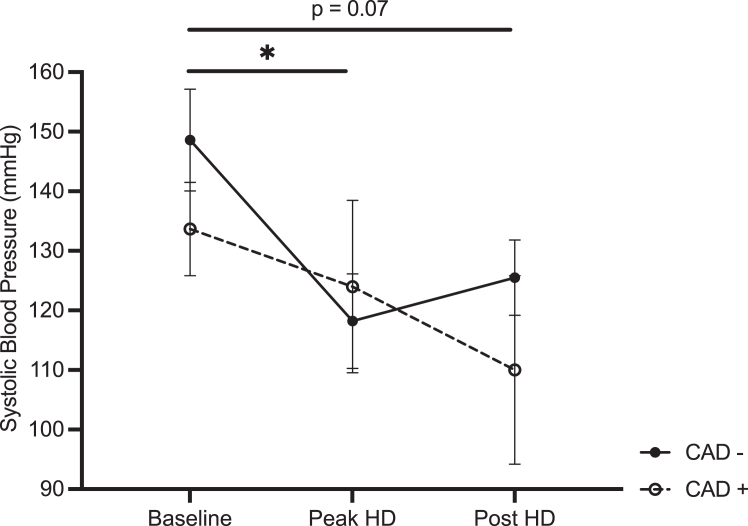
Mean systolic blood pressure in *CAD−* (n = 10) and *CAD+* arm (n = 3) at baseline, peak HD stress, and post-HD. Error bars represent the standard error of the mean. Solid significance bar is based on the post hoc test performed on the nonstenosed group. CAD, coronary artery disease; HD, hemodialysis. ∗Denote *P* < 0.03.

A significant effect of intradialytic timepoint on SBP was demonstrated, specifically in the CAD− group from baseline to peak HD timepoint with a mean difference of 30.4 mm Hg and 95% CI (2.0-58.8) (Fig 2).

### Global Myocardial Perfusion

**Figure 3:**
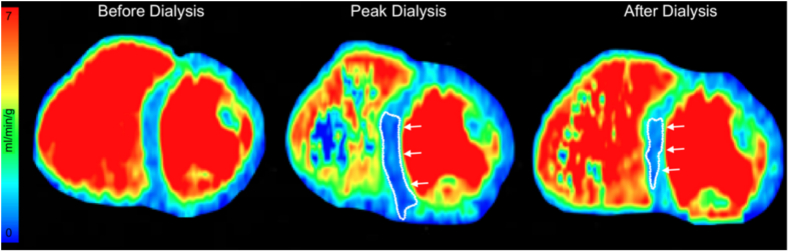
Qualitative assessment of changes in myocardial perfusion through dialysis of a single participant, in the absence of coronary artery stenosis. Cardiac image in of a short axis view and the regions outlined in white and identifiable by the white arrows represent myocardial regions with perfusion reduction.

**Figure 4:**
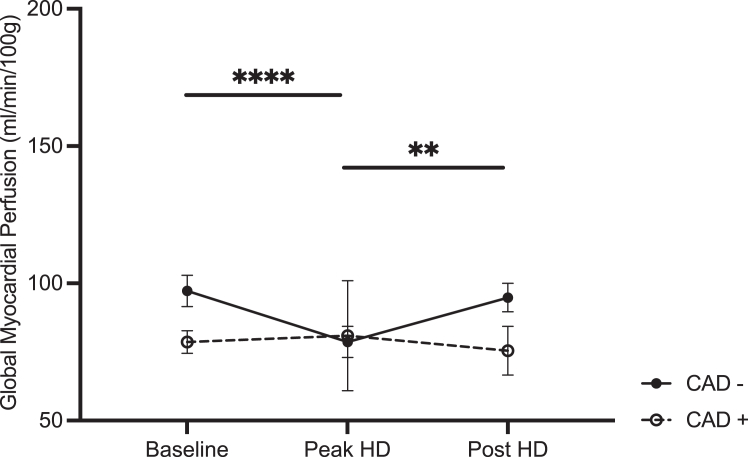
Mean global myocardial perfusion in *CAD−* (n = 10) and *CAD+* (n = 3) participants. Error bars represent the standard error of the mean. Significance bar for pairwise comparison reflects that within the nonstenosed group. ∗∗Denote *P* < 0.002. ∗∗∗∗Denote *P* < 0.001.

There was no significant effect of group (CAD+ vs CAD−) on global myocardial perfusion. Fig 3 qualitatively demonstrates the effect of timepoint on a single participant. Additionally, there was a trend toward an effect of timepoint in the CAD− group, demonstrating a mean difference in perfusion of 18.6 mL/min/100 g with a 95% CI (12.0-25.2) from baseline to peak HD. Followed by an increase in perfusion at post-HD compared with peak HD (mean difference of −16.2 mL/min/100 g with 95% CI [−25.8 to −6.5], Fig 4). There were no significant changes in intradialytic global MP measurements in the CAD+ participant group (Fig 4).

### Regional Wall Motion Abnormality

**Figure 5:**
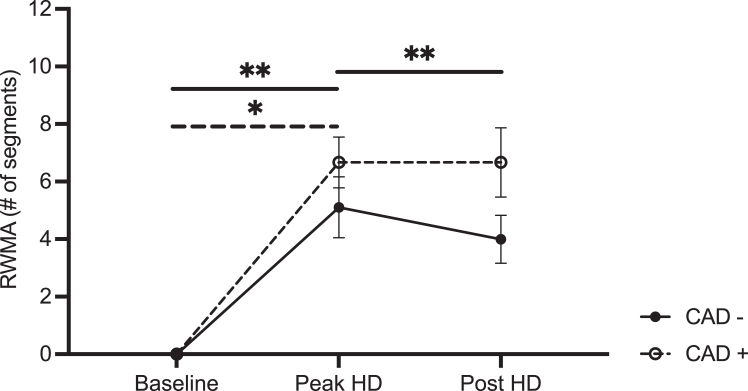
Mean regional wall motion abnormality in *CAD−* (n = 9) and *CAD+* (n = 3) participants. Error bars represent the standard error of the mean. Solid significance bars for pairwise comparison performed in the nonstenosed group. Dotted significance bar for pairwise comparison completed for the stenosed arm. ∗Denote *P* < 0.03. ∗∗Denote *P* < 0.002.

A significant effect of timepoint on RWMA was demonstrated with no significant group effect (CAD+ vs CAD−) (Fig 5). There was an increase in segments experiencing RWMAs in the CAD− participant group from baseline to peak HD (mean difference of −5.1 segments with 95% CI [−8.1 to −2.0]), that was reduced post-HD compared with peak HD (mean difference of 1.1 segments with 95% CI [−1.3 to 3.6]). In the CAD+ group, there was a similar increase in segments experiencing RWMAs at peak HD relative to baseline (mean difference of −6.7 segments with 95% CI [−11.9 to −1.5]). A trend toward increased segments experiencing RWMA at post-HD relative to baseline (mean difference of 0 segments with 95% CI [−3.4 to 3.4]) was also detected for the CAD+ group.

## Discussion

This study demonstrated HD-induced circulatory stress, as measured by a reduction in MP and development of myocardial stunning, occurs in the absence of CAD. It was revealed that almost a quarter of our study population had considerable flow-limiting plaque burden, despite having no symptoms of CAD, and there was an interaction between large vessel CAD and HD-induced myocardial stunning. These stenoses aggravate perfusion anomalies in their associated myocardial segments, resulting in the development of RWMAs that did not resolve immediately after HD.

Participants without CAD experienced a significant increase in myocardial stunning during HD, which appears to be related to the reduction in MP. This is in line with previous findings, demonstrating intradialytic reduction in myocardial perfusion during HD. Our group has previously demonstrated a reduction in regional MP throughout dialysis using positron emission tomography, with its lowest mean perfusion measurement at 4 hours.^37^ They also determined that myocardial segments identified to have RWMAs were significantly associated with greater reduction in MP from baseline when compared with normal segments.^37^ This study also identified a greater than 30% reduction in perfusion from baseline as the perfusion threshold associated with regional wall motion abnormality.^37^ It is evident from our study that with HD, there is a general reduction in global MP and an increased number of myocardial segments experiencing RWMAs in participants with no CAD. Shortly after the end of HD, global MP is recovered and stunned myocardial segments are partially salvaged on reperfusion.

The addition of CAD appears to be associated with a worsened response to HD, as evidenced by the consistently hypoperfused myocardium and prolonged stunning that does not recover following the end of HD. We have shown that unlike the traditional perfusion response seen with HD, participants with CAD showed no intradialytic perfusion changes; however, mean perfusion measurements tended to be lower at baseline and post-HD timepoint when compared with those without known CAD. The modest level of global MP in the CAD+ arm at baseline may be the result of flow-limiting epicardial artery stenoses identified through coronary angiography. In all intradialytic timepoints, myocardial segments corresponding to coronary arteries with stenosed lesions greater than 50% demonstrated trends of lower perfusion, indicating that these stenoses were in fact flow-limiting (Fig S2). The reduction in perfusion in myocardial segments affected by CAD was most evident after the end of HD. This suggests that reperfusion of these segments may take longer to return to baseline levels, which is also evident through the lack of improvement in the number of myocardial segments with greater than 30% reduction in perfusion in CAD+ arm (Fig S3). With this in mind, it is interesting to see that with the extended duration of ischemic-reperfusion injury associated with CAD, the number of stunned myocardial segments remained elevated and persistent from peak HD to post-HD. These findings suggest that CAD worsens the myocardial response to HD, potentially in aspects of the severity of RWMA and the recovery phase of HD (post-HD). Specifically, CAD may influence how quickly the stunned segment transits from hibernation to fixed nonrecoverable fibrosis.

Laboratory measurements that have been important predictors of CAD in patients treated with HD include cTnT and CRP. An increase in cTnT has been used as a marker of myocardial cell injury in clinically suspected myocardial ischemia.^44^ There continues to be controversy regarding the clinical usefulness of cardiac troponin levels to diagnose acute ischemic injury in the chronic HD population, because its levels are frequently higher at baseline without evidence of injury.^45^^,^^46^ In this study, there were no evident intradialytic changes in mean cTnT levels in the 13participants treated with HD, despite its higher-than-normal levels. The 3 participants with CAD showed trends of increased cTnT at baseline relative to the CAD− arm that remained higher at peak HD and post-HD timepoints. The elevated levels of cTnT at baseline in the CAD+ arm may represent the existence of a prior silent infarct or subclinical myocardial ischemia in which the stenosis constructs the delivery of blood to the myocardial cells, causing injury. The presence of a coronary stenoses may potentially have an additive effect on cTnT levels in those with chronic HD. The lack of change in troponin levels throughout dialysis in the CAD+ arm may be a response to the increased ischemia experienced in these participants when HD-induced and stenoses effects are combined.

One study completed on a HD population concluded that the probability of cardiovascular disease almost doubles when the serum CRP levels are greater than 0.6 md/dL.^47^ The CRP shows trends of higher concentration in the blood stream of the CAD+ group than the nonstenosed. Generally, CRP > 3.0 mg/L is a major risk factor for heart disease.^48^ In this study, the mean CRP levels at baseline was >3.0 mg/L in HD with no CAD. This elevation in CRP in CAD− participant treated with HD can be a result of the chronic inflammation associated with HD that is most evident with significant intradialytic changes in CRP. The mean CRP levels are approximately doubled in participants on HD with CAD. The CRP levels has shown to be a possible biomarker of vascular inflammation in CAD.^49^^,^^50^ The excessive levels of CRP we see in the CAD+ arm may be an indication of vascular inflammation and vessel damage not only localized to the affected artery but may also reflect ischemia in other vascular beds associated with HD.

This study was an exploratory study with a limited number of participants treated with HD; data should be interpreted with this in mind. Despite the small number of participants, the effect sizes of the reported results in the present study are large. Cohen d was calculated for the primary outcomes of global MP and RMWAs. The calculated Cohen d value for global MP between the 2 arms at baseline, peak HD, and post-HD were 1.38, 0.08, and 1.21, respectively. The calculated Cohen d value for regional wall motion abnormality between the 2 arms at peak HD and post-HD were 0.62 and 1.16, respectively. Generally, a Cohen d value greater than 0.8 is considered to be a large effect size. Although the number of patients studied was small, it was able to demonstrate the circulatory and cardiac functional changes to HD with respect to coronary artery health. It is important to also note that as a result of the small sample size, it is unlikely that other variables presented in this work were powered to detect change.

HD characteristically results in challenged myocardial perfusion that is not specific to the presence of CAD. However, this study demonstrates that concomitant CAD is associated with significant and more persistent RWMA development. Based on these findings, there is an urgent need to better understand the interaction and further justify coronary imaging to modify the natural history of HD-related cardiomyopathy. This will allow for improved outcomes in both dialysis and subsequently transplanted patients.

## Supplementary Materials

Supplementary File (PDF) Figures S1-S5; Item S1.
