# Supplementary material for: Coronary Artery Disease and Intradialytic Myocardial Ischemia in Hemodialysis: An Exploratory Study Using Intradialytic Imaging
Source: Kidney Med. 2025 Sep 24;7(12):101126. doi: 10.1016/j.xkme.2025.101126 (PMC12671370; doi:10.1016/j.xkme.2025.101126)
Supplement: Supplementary File (PDF) — Figures S1-S5; Item S1. [file mmc1.pdf]

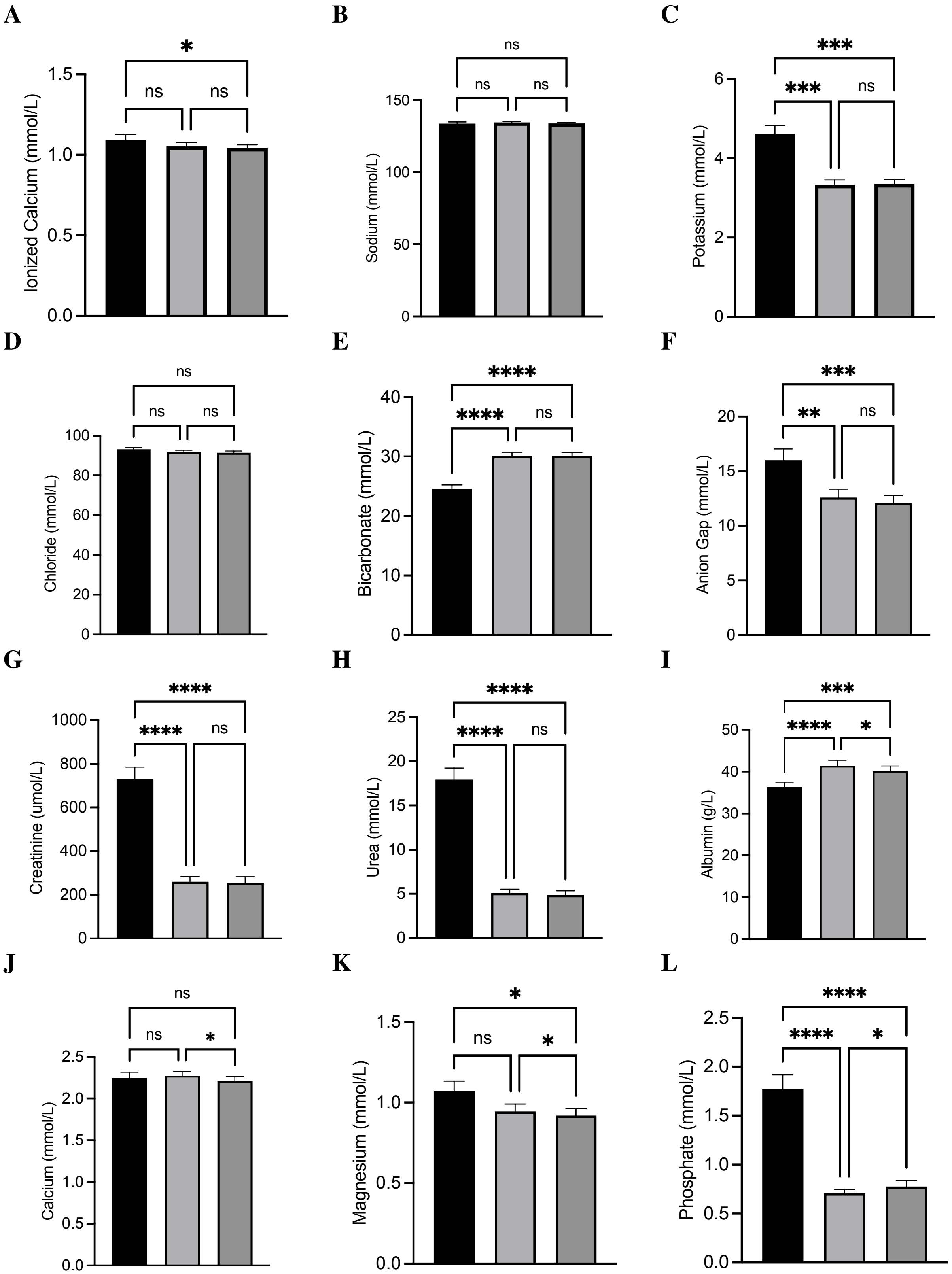
A
Ionized Calcium (mmol/L)
*
ns
ns
B
Sodium (mmol/L)
ns
ns
ns
C
Potassium (mmol/L)
***
***
ns
D
Chloride (mmol/L)
ns
ns
ns
E
Bicarbonate (mmol/L)
****
****
ns
F
Anion Gap (mmol/L)
***
**
ns
G
Creatinine (umol/L)
****
****
ns
H
Urea (mmol/L)
****
****
ns
I
Albumin (g/L)
***
****
*
J
Calcium (mmol/L)
ns
ns
*
K
Magnesium (mmol/L)
*
ns
*
L
Phosphate (mmol/L)
****
****
*

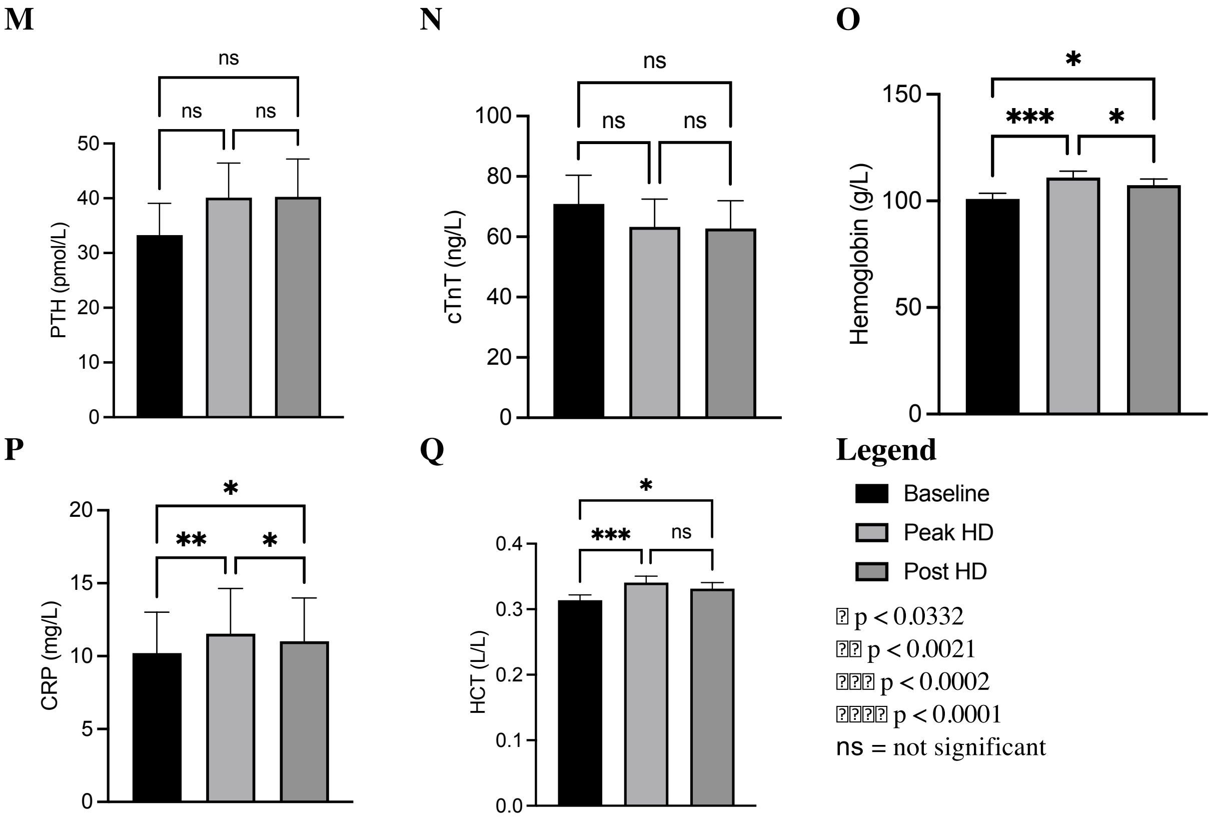

Figure S1 Intradialytic changes in serum electrolyte and protein levels. Error bars represent standard error of the mean.

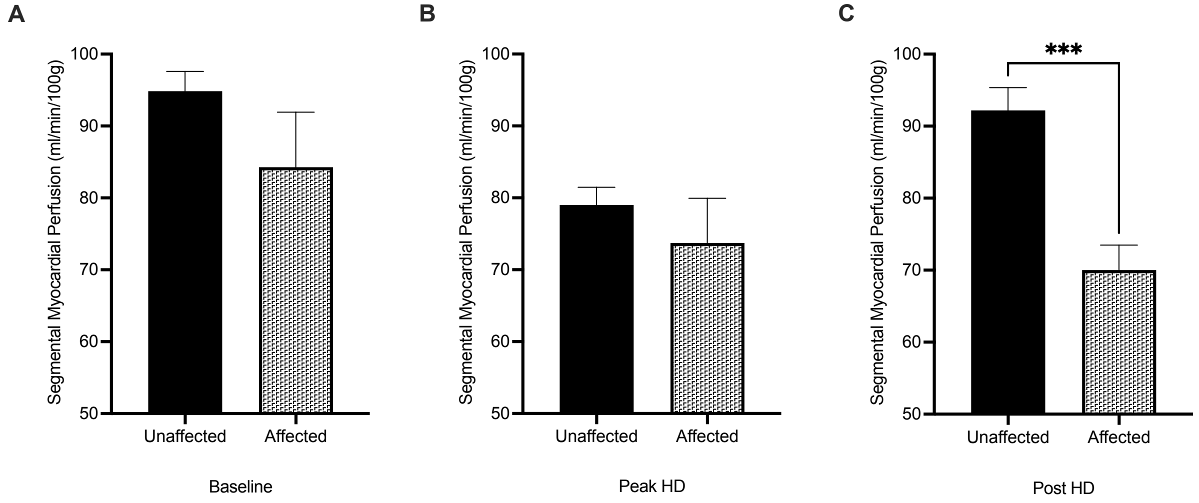

Figure S2 Mean myocardial perfusion in unaffected segments (n=156) and affected segments (n=39) at (A) baseline, (B) peak HD stress, and (C) post HD. Error bars represent the standard error of the mean. *** denote significance of $p < 0.0002$.

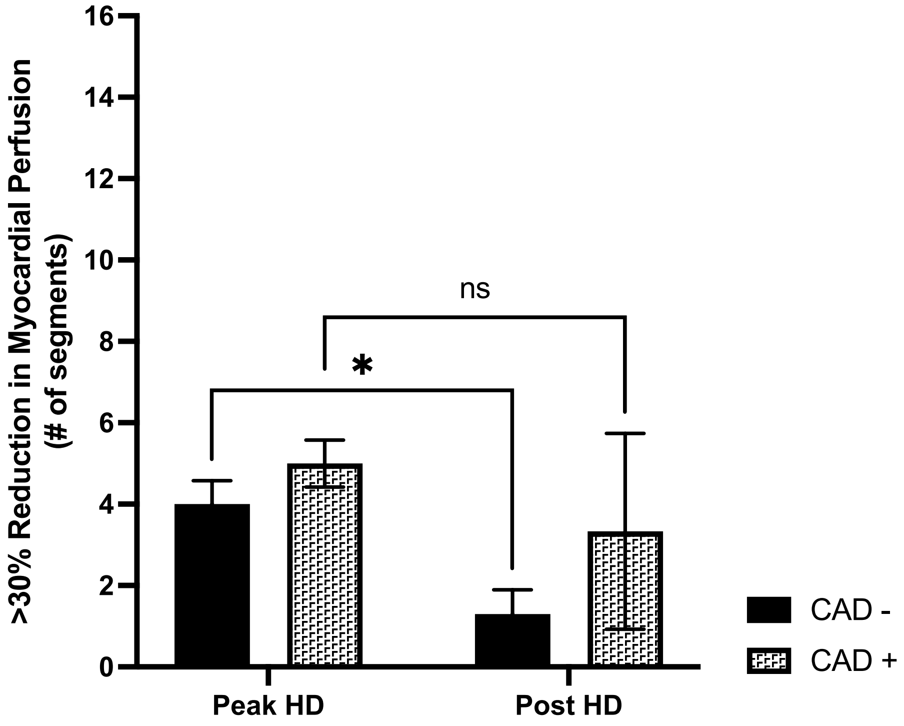

Figure S3 Mean number of myocardial segments with greater than 30% reduction in myocardial perfusion at peak HD stress and post HD relative to baseline myocardial perfusion. Error bars represent standard error of the mean. * denote $p < 0.03$.

Item S1: Supplementary Methods

Statistical Analysis

All statistical analyses presented in this paper were performed in GraphPad Prism 9 software (GraphPad Software, La Jolla, California, United States of America).

Participant Demographics and Dialysis Treatment Information

Demographic and dialysis treatment information for the thirteen participants are summarized with descriptive statistics (minimum value, maximum value, mean and standard deviation) in Tables 1 and 2.

Assessment of Hemodynamic Stability

Hemodynamic stability was assessed using systolic blood pressure measurements. To determine differences in systolic blood pressure between the *CAD*+ and *CAD*- arm, grouped analysis was performed. A repeated measure two-way ANOVA with group (*CAD*+ and *CAD*-), timepoint (baseline, peak HD, or post HD), and group-by-timepoint as fixed effects was performed for the systolic blood pressure measurements. A Geisser-Greenhouse correction was applied. If any of the fixed effects were statistically significant, post-hoc tests were performed with Sidak's correction for multiple comparisons to determine systolic blood pressure differences between intradialytic timepoints and between groups.

Laboratory Testing

Intradialytic changes in electrolyte concentrations measured from the blood samples were assessed using linear mixed models. Specifically, levels of ionized calcium, sodium, potassium, chloride, bicarbonate, anion gap, creatinine, and calcium were analyzed using linear mixed models with intradialytic timepoint as the fixed effect and participant as the repeated effect. A maximum likelihood algorithm was used to estimate variance parameters. The Geisser-

Greenhouse correction was applied to correct for the sphericity of the data. If there was a significant effect of timepoint, post-hoc testing was completed with Tukey's correction for multiple comparison to identify differences in electrolyte between timepoints. A linear mixed model was used for these data, as some participants did not have measures of all electrolytes at all timepoints.

In contrast, intradialytic changes in urea, albumin, magnesium, phosphate, parathyroid hormone, cardiac troponin T, hemoglobin, C-reactive protein, and hematocrit levels were measured for all participants at all timepoints. As such, these data were analyzed using repeated measures one-way ANOVA. The Geisser-Greenhouse correction was applied to correct for the sphericity of the data. If the ANOVA was statistically significant, post-hoc testing with Tukey's correction for multiple comparison was completed to determine differences between timepoints.

Grouped analysis was performed for cardiac troponin T and C-reactive protein measurements. A repeated measure two-way ANOVA test with intradialytic timepoint and group (*CAD+* and *CAD-*) as fixed effects were performed with the Geisser-Greenhouse correction for sphericity. If any fixed effect was statistically significant, post-hoc testing was completed with Sidak's correction for multiple comparisons to determine differences between groups and between timepoints.

Global Myocardial Perfusion

A grouped analysis was performed to investigate the effect of coronary artery disease on global MP during dialysis. A linear mixed model with group (*CAD+* and *CAD-*) and intradialytic timepoint as fixed effects was performed with restricted maximum likelihood estimation and the Geisser-Greenhouse correction. Post-hoc testing with Tukey's correction for multiple comparison was performed to identify differences in MP within and between groups.

Segmental Myocardial Perfusion

Segmental MP measurements from all participants were pooled for analysis. Myocardial segments perfused by coronary arteries identified with greater than 50% stenoses were grouped together while myocardial segments perfused by non-stenosed coronary arteries composed another group. In total, 156 myocardial segments received their blood supply from normal coronary arteries ('unaffected' group) and 39 myocardial segments were supplied by coronary arteries that had stenosed lesions ('affected' group). At each individual timepoint (baseline, peak HD, post HD), a parametric unpaired two-tailed t-test was performed to determine statistical significance in MP between the unaffected and affected group.

The number of myocardial segments with greater than thirty percent reduction in perfusion relative to baseline perfusion values was determined at peak HD and post HD. A repeated measure two-way ANOVA test with intradialytic timepoint and group (*CAD*+ and *CAD*-) as fixed effects was performed with the Geisser-Greenhouse correction for sphericity. If any fixed effect was statistically significant, post-hoc testing was completed with Sidak's correction for multiple comparison to determine differences between groups and between timepoints.

Regional Wall Motion Abnormality

The number of myocardial segments experiencing RWMAs was analyzed with a repeated measure one-way ANOVA. If the ANOVA demonstrated statistical significance, a post-hoc testing was completed with Tukey's correction for multiple comparisons to identify differences between intradialytic timepoints.

Additionally, a grouped analysis was performed to investigate the effect of coronary artery disease on the number of myocardial segments experiencing RWMAs during dialysis. A repeated measure two-way ANOVA was conducted with group (*CAD*+ and *CAD*-) and

intradialytic timepoint as fixed effects and the participant as the repeated effect. A Geisser-Greenhouse correction was applied for this analysis. If any of the fixed effects were significant, post-hoc tests were completed with Tukey's correction for multiple comparisons to identify differences in the number of myocardial segments experiencing RWMAs within and between groups.

Supplementary Results

Segmental Myocardial Perfusion

At baseline (Figure S2A), the segmental MP measurements were not significantly different between the affected and unffected group (mean difference of -10.6 ml/min/100g with 95% CI [-23.8,2.6]). Similarly, the segmental perfusion measurements were not different between groups at peak HD (Figure S2B, mean difference of -5.3ml/min/100g with 95% CI [-16.8,6.3]). At post HD timepoint (Supplementary Figure 2C), the mean segmental MP in the affected group was significantly lower compared to the unaffected group (mean difference of -22.2 ml/min/100g with 95% CI [-35.1,9.2]).

The number of myocardial segments with >30% reduction in MP were quantified (Figure S3), showing no significance in the interaction between timepoint and group. However, post-hoc testing showed that fewer myocardial segments experienced >30% reduction in perfusion at post HD compared to peak HD in *CAD-* participant group ($p = 0.05$). In participants with *CAD+*, no statistical difference between the timepoints was seen ($p = 0.60$).
